# Sulfotanshinone Sodium Injection for Unstable Angina Pectoris: A Systematic Review of Randomized Controlled Trials

**DOI:** 10.1155/2012/715790

**Published:** 2012-04-04

**Authors:** Xuelan Qiu, Andrew Miles, Xuehua Jiang, Xin Sun, Nan Yang

**Affiliations:** ^1^Department of Clinical Pharmacy & Pharmacy Administration, West China Pharmacy School, Sichuan University, No. 17 Section 3 Renmin Nanlu, Chengdu, Sichuan 610041, China; ^2^Department of Acupuncture & Chinese Medicine, Canadian College of Acupuncture and Oriental Medicine, 551 Chatham Street, Victoria, BC, Canada V8T 1E1; ^3^Center for Health Research North West, Kaiser Permanente, 5025 Southeast 28th Avenue, Portland, OR 97202, USA; ^4^Department of Clinical Epidemiology and Biostatistics, McMaster University, Hamilton, ON, Canada L8S 4L8

## Abstract

*Objective*. To assess the effect of sulfotanshinone sodium injection for unstable angina. *Methods*. We searched for published and unpublished studies up to June 2011. We included randomized controlled trials that confoundedly addressed the effect of sulfotanshinone sodium injection in the treatment of unstable angina. *Results*. Twenty-five studies involving 2,377 people were included. There was no evidence that sulfotanshinone sodium alone had better or worse effects to routine western medicine treatments in improving clinical symptoms (RR 1.00, 95% CI 0.90 to 1.11) and ECG (RR 0.97, 95% CI 0.87 to 1.09). However, there was evidence that sulfotanshinone sodium combined with western medications was a better treatment option than western medications alone in improving clinical symptoms (RR 1.28, 95% CI 1.23 to 1.3), ECG (RR 1.26, 95% CI 1.18 to 1.35), C-reaction protein (mean difference 2.10, 95% CI 1.63 to 2.58), and IL-6 (mean difference −3.85, 95% CI −4.10 to −3.60). There was no difference between sulfotanshinone sodium plus western medications and western medications alone affecting mortality (RR 0.50, 95% CI 0.02 to 12.13). *Conclusion*. Compared with western medications alone, sulfotanshinone sodium combined with western medications may provide more benefits for patients with unstable angina. Further large-scale high-quality trials are warranted.

## 1. Introduction

Coronary artery disease is the leading cause of death in the United States [1]. Early hospital care for unstable angina includes anti-ischemic therapies, antiplatelet therapies, and anticoagulant/antithrombotic therapies and may also consider an early invasive strategy [2]. Thrombolytic agents are usually more frequently used for more severe conditions [3–5].

Danshen*, also known as Salvia miltiorrhiza Bge*, is a hemorheologic agent that may have protective effect in patients with unstable angina [6] and has been used for cardiovascular disorders for hundreds of years in China and now is widely used in other countries as well.

Danshen consists of a mixture of compounds, among which Tanshinone IIA (TIIA) represents the most biologically active ingredient [7]. TIIA, also known as Danshen ketone, Tanshinon II, Tanshinone B, is a diterpenoid naphthoquinone extracted and isolatedderivative from Danshen. Animal and cellular studies have shown various potential benefits of the agent, including (1) neuroprotective effect in cerebral ischemia and reperfusion [8], (2) antioxidant potential to prevent oxidation of low-density lipoproteins [9], (3) ability of rescuing PC-12 cells from hypoxia [10], (4) reducing cellular damage by free radicals [11], (5) protecting mitochondrial membrane from ischemia-reperfusion injury and lipid peroxidation [12], (6) decreasing PHB expression in oxidative stress-injured myocardial cells hence protecting the myocardial cells [13], (7) protecting cardiomyocytes against oxidative stress-mediated apoptosis [14], and (8) cardioprotective in the context of diabetic cardiomyopathy through kinin B2 receptor-Akt-GSK-3*β*-dependent pathway [15]. Human studies also have demonstrated cardioprotective effects of TIIA, including reduction of myocardial infarct size and decrease of myocardial consumption of oxygen [16].

Until now, the clinically available TIIA agent, which is approved by State Food and Drug Administration of China, only includes sulfotanshinone sodium (SS) injection (i.e., sodium tanshinone IIA sulfonate injection) manufactured by two companies. TIIA is extracted from the raw herb Danshen and then chemically derivatized into water-soluble SS for the preparation of injection. Upon the administration of SS injection, SS transforms back into the bioactive ingredient TIIA in vivo [17]. The dosage for administration of SS injection is 40–80 mg per day. SS injection is given diluted at the point of treatment in 20 mL 25% glucose injection for intramuscular administration or in 250–500 mL 5% glucose injection for intravenous administration. It is widely used in the Chinese hospitals for unstable angina [18].

However, the effects of SS injection on unstable angina have not been well established. In this study, we evaluated the effect of SS through a rigorous systematic review and meta-analysis of randomized trials.

## 2. Methods

### 2.1. Eligibility Criteria

We included randomized controlled trials that compared SS with placebo or active agents in patients with unstable angina defined as new onset (≤2 months) exertional angina of at least Canadian Cardiovascular Society Classification (CCSC) class III in severity, significant recent increase in frequency and severity of angina, or angina at rest.

The eligible comparisons include

- SS injection versus any current western medications,
- SS injection plus any current western medications for unstable angina versus western medications alone,
- SS injection versus placebo.

Our prespecified primary outcome is all-cause mortality, and secondary outcomes include resolution of angina, ECG improvement, inflammatory factors (such as C-reaction protein and IL-6), and adverse events. The improvement of clinical symptoms is measured as the reduction in chest pain and shortness of breath or the frequency, severity, and length of acute angina attacks. “Very effective” includes that there is no angina attack, chest pain disappears, ST segment depression is back to normal, or the depression of ST segment reduces >0.1 mV; “effective” includes that times of angina attacks reduce by >2/3 or the length, frequency, and severity of angina attacks and chest pain significantly reduce, the depression of ST segment reduces <0.1 mV but >0.05 mV; “ineffective” includes that there is no change or very little change in chest pain and shortness of breath, or the frequency, severity and length of acute angina attacks, the depression of ST segment reduces <0.05 mV. For the systematic review, the outcomes of both “very effective” and “effective” were considered successful treatments.

### 2.2. Search Strategy

We searched the Cochrane Library (Issue 7, 2011), Chinese Cochrane Centre Controlled Trials Register (to June 2011), Medline (1995 to June 2011), EMBASE (1995 to June 2011), CNKI database (1979 to June 2011), Wanfang Data (1998 to June 2011), and VIP Information (1985 to June 2011) using the following key words: unstable angina, angina, SS, tanshinone IIA, and sodium tanshinone IIA sulfonate, as well as the brand names of the agent. We also searched databases of ongoing trials, including Current Controlled Trials and the UK National Research Register.

We also searched Chinese medicine journals not indexed in the electronic databases. We screened the reference lists of relevant trials and identified reviews. We contacted experts in this field and relevant pharmaceutical companies for additional references or unpublished studies. We restricted the language of publications to English and Chinese.

### 2.3. Data Collection

Two reviewers (Qiu and Yang) independently screened the titles, abstracts, and key words of each searched article for potentially eligible studies. Reviewers then screened full texts for final eligibility. The full-text articles were retrieved for further assessment if the information given suggests that the study: (1) included patients with unstable angina, (2) compared SS injection with western medication in the presence or absence of cointerventions in both groups, (3) assessed one or more relevant clinical outcome measure such as morality, clinical symptoms, or electrocardiogram (ECG), (4) had clearly outlined criteria for successful treatment and treatment success was not measured in terms of illness severity scores or the intensity of individual symptoms, and (5) used random allocation.

Reviewers independently extracted data from eligible studies, using pilot-tested data extraction forms. Reviewers extracted the following data: age and number of participants in the SS group and the control group, male-female ratio in each group, diagnosis criterion, treatment dosage and duration, side effects, and symptoms that improved after treatments. Important missing data were obtained by contacting article authors whenever possible.

We excluded studies if they (1) included nonunstable angina, used or compared with other Chinese Medicine, (2) used different routine western medications in the trial group and control group, (3) were not randomized trials, (4) had unclear criteria of symptom improvement or data error, (5) were duplicated, or (6) were not conducted on human subjects.

### 2.4. Risk of Bias

Two reviewers (Qiu and Yang) independently assessed the risk of bias for each trial according to the Cochrane Handbook for Systematic Reviews of Interventions version 5.1.0 [44]. The items included the random sequence generation, allocation concealment, blinding, incomplete outcome data, selective outcome reporting, and other potential threats to validity. Summary assessments of the risk of bias for important outcomes within and across studies was made. Based on Cochrane handbook [44], a study is considered at low risk of bias if there is low risk of bias for all key domains within a study; it is considered to be unclear risk of bias if unclear risk of bias is for one or more key domains within studies at unclear risk of bias across studies; it is considered to be high risk of bias if high risk of bias for one or more key domains within a study is sufficient to affect the interpretation of results across the studies. Disagreements were resolved by discussion and by adjudicated by a third reviewer (Jiang) when necessary.

### 2.5. Data Analysis

Our comparisons included SS versus western medication and SS plus routine therapy versus routine therapy. We reported risk ratio (RR) and 95% confidence intervals (CI) for the pooled binary data, and mean differences (MD) for continuous data. The test of homogeneity was used with a significance level of 0.1. We also used the I-square statistic to assess the heterogeneity. Publication bias was assessed by the funnel plot. We performed sensitivity analysis by using different statistical methods (fixed-effect and random-effects models) for combining data to explore the influence of study quality on effect size.

## 3. Results

**Figure 1:**
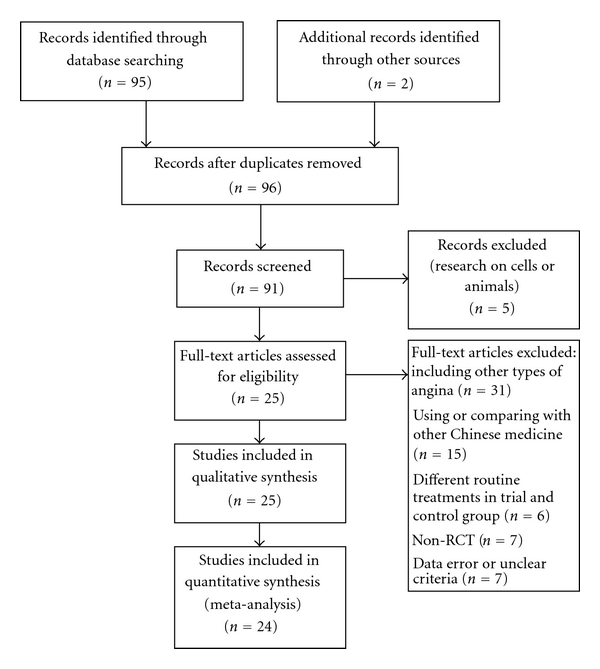
PRISMA flow chart of literature retrieval and selection.

A total of twenty-five trials [19–43], involving 2,377 participants with unstable angina defined as new onset (≤2 months) exertional angina of at least Canadian Cardiovascular Society Classification (CCSC) class III in severity, significant recent increase in frequency and severity of angina, or angina at rest, proved eligible (Figure 1).

All 25 trials were conducted in China. The treatment duration ranged from 1 to 4 weeks, and the dose from 40 to 80 mg per day. One trial [34] compared SS injection versus isosorbide mononitrate. The other 24 trials compared SS injection plus western medications versus western medications alone. There were no placebo controlled studies. SS injection is given diluted at the point of treatment in 20 mL 25% glucose injection for intramuscular administration or in 250–500 mL 5% glucose injection for intravenous administration. The dosage and administration are not clearly described in every study (Table 1).

Two trials [33, 35] reported data on mortality. Most trials reported improvement of clinical symptoms and ECG.

All studies were at high risk of bias (Table 2). One trial [33] described the method of randomization in detail, and the method was also appropriate. All the other studies did not report information on the allocation concealment. One trial [37] mentioned it is a single-blinded study, and none were double blinded. Loss to followup was recorded in none of the studies. No studies conducted intention-to-treat analysis.

### 3.1. SS versus Western Medications

**Figure 2:**
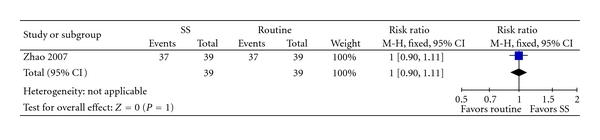
SS versus Isosorbide, outcome: clinical symptom improvement.

**Figure 3:**
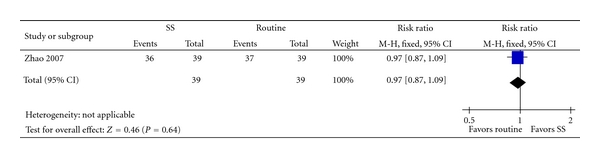
SS versus Isosorbide, outcome: ECG.

One trial [34] compared SS alone versus western medicine. There were no significant differences in improvement of clinical symptoms (RR 1.00, 95% CI 0.90 to 1.11, Figure 2) and improvement in ECG (RR 0.97, 95% CI 0.87 to 1.09, Figure 3).

### 3.2. SS + Western Medications versus Western Medications

Two trials [33, 35] comparing SS plus western medications versus western medications reported only one sudden death in the western medication group [33] (RR 0.50; 95% CI 0.02 to 12.13).

**Figure 4:**
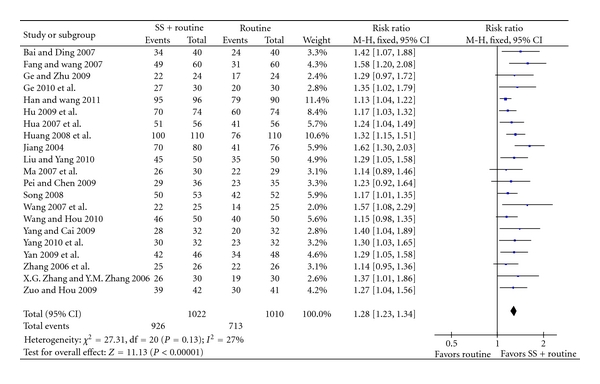
SS + routine therapy versus routine therapy, outcome: clinical symptom improvement.

**Figure 5:**
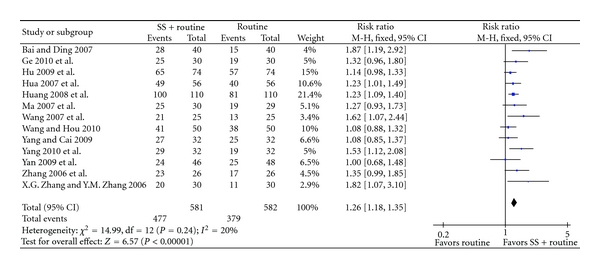
SS + routine therapy versus routine therapy, outcome: ECG.

**Figure 6:**
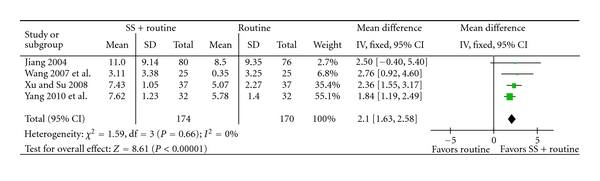
SS + routine therapy versus routine therapy, outcome: C-reaction Protein.

**Figure 7:**
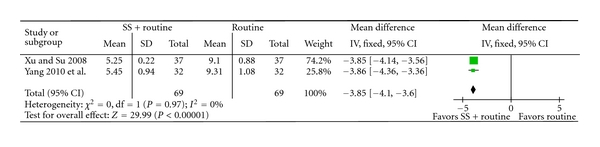
SS + routine therapy versus routine therapy, outcome: IL-6.

Sodium plus western medications achieved statistically significant improvement of clinical symptoms than western medications alone (RR 1.28, 95% CI 1.23 to 1.34, Figure 4), and improvement of ECG (RR 1.26, 95% CI 1.18 to 1.35, Figure 5), C-reaction protein (mean difference 2.10, 95% CI 1.63 to 2.58, Figure 6), and IL-6 (mean difference −3.85, 95% CI −4.10 to −3.60, Figure 7).

Of the 25 trials, 7 reported adverse events. In the routine treatment group, adverse reactions included headache, dizziness, facial flushing, fatigue, and bruises at injection site. Totally 32 cases were reported. In the SS + routine treatment group, adverse reactions included facial flushing, dizziness, bruises, tension or swell at injection site, blood in sputum, and gum bleeding. Totally 13 cases were reported. No severe adverse events were found and no treatment was stopped because of adverse events.

**Figure 8:**
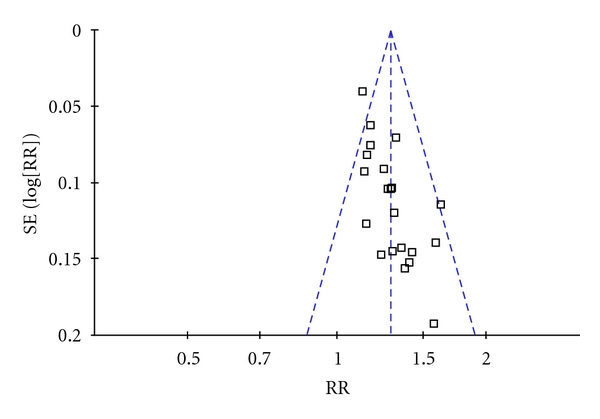
Funnel plot of comparison: SS plus routine therapy versus routine therapy, outcome: clinical symptom improvement. (Each dot represents one study. All the dots are conforming to a triangular form, meaning that publication bias is low).

Because the funnel plot seems symmetric, the possibility that the result of this review might be misled by publication bias is likely to be little (Figure 8).

## 4. Discussion

SS injection appeared an effective and safe treatment option for unstable angina pectoris. The present results showed that SS plus routine therapies appear to be more effective than western medications alone.

However, trials are at high risk of bias, making the findings less compelling. Except for one trial, none of the other trials reported the method of randomization. Although all trials claimed randomization, they failed to provide enough information to judge whether the randomization procedures had been carried out properly. No multicenter, large-scale RCTs were identified. No dropouts and withdrawals were described. No placebo control was used and none of the trials were of double blind. Routine therapy varied from trial to trial. The dosage and administration of control and trial therapies are not clearly described in every study.

The main outcomes from the included 25 trials were the improvement of clinical symptoms and ECG. The primary outcome measure was reported in only two trials. There is lack of data from RCTs on clinically relevant outcomes from long-term followup such as mortality and health-related quality of life.

18 out of 25 trials referred to observation of side effects. There were less side events in the SS injection group. None of the events were severe and no patients dropped out because of the side effects. SS injection appears to be relatively safe.

We have conducted comprehensive searches. However, only trials published in English and Chinese were indentified. Unpublished studies were found but none of them met the inclusion criteria. Since all of the trials were of small size with positive results and were conducted China, geographic biases may be induced.

The poor evidence does not allow any conclusion regarding the effectiveness of SS, and none of the included trials were ideally suited to investigate the effectiveness of SS in treating unstable angina. While SS is a widely used therapy for unstable angina in China, the results of the present review suggest that high-quality controlled trials are required for assessment.

## 5. Conclusions

Compared with western medications alone, SS combined with western medications was of more benefits for patients with unstable angina with fewer side effects. However, the methodological concerns, such as allocation concealment, lack of blinding, lack of information on the hazards of treatment, and the risk of publication bias, make it difficult to determine the role of SS injection in management of unstable angina.

Considering the strength of the evidence, more rigorously designed, randomized double-blind placebo-controlled trials are required for assessing the effects of SS injection before SS injection can be recommended routinely. Some aspects should be specially considered, including methodological improvement (such as details on the methods of randomization and the allocation concealment, blinding and placebo control, dropouts and withdrawals), adverse reactions, and reporting clinically outcomes from long-term followup such as mortality and health-related quality life.

## Figures and Tables

**Table 1:** Randomized controlled trials of SS injection for unstable angina pectoris.

Study	Method	N (M : F)	Mean age	Interventions	Outcomes
Zhao 2007 [19]	RCT, not blinded Duration: 2 W	78 (54 : 24)	62.8	(1) Isosorbide mononitrate 40 mg (2) SS 40 mg	(1) clinical symptom improvement, (2) ECG, (3) frequency, duration and intervals of angina attacks
Yan et al. 2009 [20]	RCT, not blinded Duration: 4 W	94 (53 : 41)	52	(1) Routine (Aspirin 300 mg–100 mg qd, Enoxaparin, Elantan 50 mg, Betaloc 100 mg) (2) Routine + SS 60 mg	(1) clinical symptom improvement, (2) ECG, (3) FIB, (4) D-dimer
Wang and Hou 2010 [21]	RCT, not blinded Duration: 2 W	100 (65 : 35)	62	(1) Routine (Aspirin, Nitrates, Calcium antagonists, Ozagrel) (2) Routine + SS 40 mg	(1) clinical symptom improvement, (2) ECG
Yang et al. 2010 [13, 22]	RCT, not blinded Duration: 1 W	64 (35 : 39)	59	(1) Routine (Aspirin 100 mg qd, Isosorbide mononitrate 20 mg bid, Metoprolol 25 mg bid) (2) Routine + SS 60 mg	(1) clinical symptom improvement, (2) ECG, (3) C-reaction protein, (4)IL-6, (5) plasma viscosity, (6) FIB
Ge et al. 2010 [23]	RCT, not blinded Duration: 15 D	60 (39 : 21)	58	(1) Routine (Nitrates, Betaloc, anticoagulant and antiplatelet aggregation medication, ACEI, Statins) (2) Routine + SS 60 mg	(1) clinical symptom improvement, (2) ECG, (3) TC, TG, LDL-C, HDL-C
Ge and Zhu 2009 [24]	RCT, not blinded Duration: 2 W	48 (32 : 16)	40–80Range	(1) Routine (Aspirin, Betaloc, ACEI, Calcium antagonists, Isosorbide mononitrate, antiplatelet agents, Trimetazidine) (2) Routine + SS 50 mg	(1) clinical symptom improvement.
Hu et al. 2009 [25]	RCT, not blinded Duration: 2 W	148	60	(1) Routine (Statins, ARB, ACEI, Nitrates, Aspirin, LMWH, Betaloc) (2) Routine + SS 40 mg	(1) clinical symptom improvement, (2) ECG.
Pei and Chen 2009 [26]	RCT, not blinded Duration: 2 W	71 (48 : 23)	65	(1) Routine (Aspirin, Clopidogrel, LMWH, Nitrates, Betaloc, Statins, nondihydropyridine calcium antagonists) (2) Routine + SS 40 mg	(1) clinical symptom improvement, (2) plasma viscosity, (3) blood viscosity at high/low shear stress, (4) hematocrit.
Zuo and Hou 2009 [27]	RCT, not blinded Duration: 2 W	83 (58 : 25)	72	(1) Routine (Aspirin, Betaloc, Nitrates, Statins, LMWH) (2) Routine + SS 40 mg	(1) clinical symptom improvement, (2) length of angina from attacking to alleviating, (3) length of angina from attacking to vanishing, (4) times of myocardial ischemia onset.
Song 2008 [28]	RCT, not blinded Duration: 2 W	105	72	(1) Routine (Aspirin, Simvastatin, Betaloc, Nitrates, Diltiazem, ARB, ACEI) (2) Routine + SS 60 mg	(1) clinical symptom improvement.
Xu and Su 2008 [29]	RCT, not blinded Duration: 1 W	74 (40 : 30)	45–78Range	(1) Routine (Fluvastatin, Aspirin, Betaloc, LMWH) (2) Routine + SS 80 mg	(1) C-reaction protein, (2) IL-6, (3) P-selectin, (4) PAI-1
Huang et al. 2008 [30]	RCT, not blinded Duration: 2 W	220 (140 : 80)	62	(1) Routine (LMWH, Betaloc, Isosorbide mononitrate, calcium antagonists, Statins, Aspirin) (2) Routine + SS 60 mg	(1) clinical symptom improvement, (2) ECG, (3) plasma/whole blood viscosity, (4) systolic/diastolic blood pressure, (5) heart rate, (6) hematocrit, (7) Platelet aggregation, (8) FIB.
Li et al. 2008 [31]	RCT, not blinded Duration: 2 W	125 (80 : 45)	62.41	(1) Routine (ACEI, vasodilator, antiplatelet agents, anticoagulants) (2) Routine + SS 60 mg	(1) NO, (2) FMD, (3) ET.
Hua et al. 2007 [32]	RCT, not blinded Duration: 2 W	112 (69 : 43)	60	(1) Routine (Aspirin, LMWH, Betaloc, Nitroglycerin, ACEI, Isosorbide mononitrate) (2) Routine + SS 80 mg	(1) clinical symptom improvement, (2) ECG, (3) plasma viscosity, (4) whole blood viscosity, (5) erythrocyte aggregation, (6) morality, (7) FIB.
Wang et al. 2007 [33]	RCT, not blinded Duration: 2 W	50 (28 : 22)	48.5	(1) Routine (Betaloc, Isosorbide mononitrate, Diltiazem, Aspirin) (2) Routine + SS 50 mg	(1) clinical symptom improvement, (2) ECG, (3) D-dimer, (4) C-reaction protein, (5) plasma viscosity, (6) erythrocyte aggregation, (7) hematocrit.
Ma et al. 2007 [34]	RCT, not blinded Duration: 2 W	59 (37 : 22)	62.7	(1) Routine (Betaloc, Aspirin, ACEI, Isosorbide mononitrate, calcium antagonists, anticoagulants,) (2) Routine + SS 40 mg	(1) clinical symptom improvement, (2) ECG, (3) morality.
X. G. Zhang and Y. M. Zhang 2006 [35]	RCT, not blinded Duration: 4 W	60 (33 : 27)	62	(1) Routine (antiplatelet agents, Nitrates, Betaloc, ACEI, Diuretics) (2) Routine + SS 60 mg	(1) clinical symptom improvement, (2) ECG, (3) systolic/diastolic blood pressure, (4) heart rate, (5) frequency and duration of angina attacks, (6) Premature ventricular contractions in 24 hours.
Zhang et al. 2006 [36]	RCT, single blinded Duration: 2 W	52	—	(1) Routine (Nitrates, Betaloc, Aspirin) (2) Routine + SS 80 mg	(1) clinical symptom improvement, (2) ECG.
Liu and Yang 2010 [37]	RCT, not blinded Duration: 2 W	100 (61 : 49)	65	(1) Routine (LMWH 6000 U q12h, Nitrates, Simvastatin 20 mg qn, Betaloc, ACEI, Aspirin) (2) Routine + SS 50 mg	(1) clinical symptom improvement.
Yang and Cai 2009 [38]	RCT, not blinded Duration: 4 W	64 (32 : 32)	49.5	(1) Routine (Captopril 25 mg qd, Betaloc 25 mg bid, Isosorbide mononitrate 40 rag qd, Aspirin 0.1 g pd, Simvastatin 25 rag, qn) (2) Routine + SS 60 mg	(1) clinical symptom improvement, (2) ECG, (3) frequency and duration of angina attacks.
Bai and Ding 2007 [39]	RCT, not blinded Duration: 4 W	80 (42 : 38)	61	(1) Routine (Nitrates, Betaloc, ACEI, antiplatelet agents, LMWH) (2) Routine + SS 80 mg	(1) clinical symptom improvement, (2) ECG.
Fang and Wang 2007 [40]	RCT, not blinded Duration: 10 D	120 (54 : 66)	74	(1) Routine (LMWH 5000 U, Nitroglycerin 10 mg) (2) Routine + SS 60 mg	(1) clinical symptom improvement.
Jiang 2004 [41]	RCT, not blinded Duration: 3 W	156 (82 : 74)	60.9	(1) Routine (ACEI, Betaloc) (2) Routine + SS 40 mg	(1) clinical symptom improvement, (2) C-reaction protein, (3) times of angina attacks daily.
Qi and Qu 2008 [42]	RCT, not blinded Duration: 2 W	68 (38 : 30)	63	(1) Routine (Nitrates, Aspirin) (2) Routine + SS 60 mg	(1) frequency of angina attacks, (2) duration of angina attacks, (3) TC, TG, HDL, LDL.
Han and Wang 2011 [43]	RCT, not blinded Duration: 2W	186 (94 : 92)	55	(1) Routine (nitroglycerin 20 mg) (2) Routine + SS 60 mg	(1) clinical symptom improvement.

RCT: randomized clinical trial; F: female; M: male; W: week(s); D: day(s); 1: control group; 2: trial group; SS: SS; LMWH: Low molecular weight heparin; ACEI: angiotensin-converting enzyme inhibitors; ARB: angiotensin receptor blocker; TC: total cholesterol; TG: Triglyceride; HDL: high density lipoprotein; LDL: low density lipoprotein; NO: nitric oxide; FMD: flow-mediated dilation; ET: endothelin; FIB: fibrinogen; qd: once per day; qn: once per night; bid: twice per day; q12 h: once every 12 hours.

**Table 2:** Assessment of risk of bias in included studies.

Study	Random sequence generation	Allocation concealment	Blinding	Incomplete outcome data	Selective reporting	Free of other bias	Summary assessments
Zhao 2007 [19]	U	U	H	U	U	H	H
Yan et al. [20] 2009	U	U	H	U	U	H	H
Wang and Hou 2010 [21]	U	U	H	U	U	H	H
Yang et al. 2010 [22]	U	U	H	U	U	H	H
Ge et al. 2010 [23]	U	U	H	U	U	H	H
Ge and Zhu 2009 [24]	U	U	H	H	U	H	H
Hu et al. 2009 [25]	U	U	H	U	U	H	H
Pei and Chen 2009 [26]	U	U	H	U	U	H	H
Zuo and Hou 2009 [27]	U	U	H	U	U	H	H
Song 2008 [28]	U	U	H	H	U	H	H
Xu 2008 [29]	U	U	H	L	U	H	H
Huang et al. 2008 [30]	U	U	H	U	U	H	H
Li et al. 2008 [31]	U	U	H	U	U	H	H
Hua et al. 2007 [32]	L	L	H	U	U	H	H
Wang et al. 2007 [33]	U	U	H	U	U	H	H
Ma et al. 2007[34]	U	U	H	U	U	H	H
X. G. Zhang and Y. M. Zhang 2006 [35]	U	U	H	U	U	H	H
Zhang et al. 2006 [36]	U	U	H	H	U	H	H
Liu and Yang 2010 [37]	U	U	H	H	U	H	H
Yang and Cai 2009 [38]	U	U	H	U	U	H	H
Bai and Ding 2007 [39]	U	U	H	H	U	H	H
Fang and Wang 2007 [40]	U	U	H	H	U	H	H
Jiang 2004 [41]	U	U	H	U	U	H	H
Qi and Qu 2008 [42]	U	U	H	U	U	H	H
Han and Wang 2011 [43]	U	U	H	H	U	H	H

L: low risk of bias, U: unclear, H: high risk of bias.
